# Biomechanical study of anterior transpedicular root screw intervertebral fusion system of lower cervical spine: a finite element analysis

**DOI:** 10.3389/fbioe.2024.1352996

**Published:** 2024-01-31

**Authors:** Senqi Ye, Jiachun Ye, Zhipeng Hou, Xinmao You, Shufeng Shen, Jihui Zhang, Liang Yu, Yongjie Gu, Wei Wang, Liujun Zhao

**Affiliations:** ^1^ Department of Spinal Surgery, Yuyao People’s Hospital, Yuyao, China; ^2^ The Affiliated Lihuili Hospital, Ningbo University, Ningbo, China; ^3^ Health Science Center, Ningbo University, Ningbo, China; ^4^ Department of Spinal Surgery, Ningbo No 6.Hospital of Ningbo University, Ningbo, China; ^5^ Urumqi DW Innovation Infotech Co., Ltd., Urumqi, Xinjiang, China

**Keywords:** cervical spine, anterior approach, pedicle screw, intervertebral fusion system, finite element analysis

## Abstract

**Background:** The cervical anterior transpedicular screw (ATPS) fixation technology can provide adequate stability for cervical three-column injuries. However, its high risk of screw insertion and technical complexity have restricted its widespread clinical application. As an improvement over the ATPS technology, the cervical anterior transpedicular root screw (ATPRS) technology has been introduced to reduce the risk associated with screw insertion. This study aims to use finite element analysis (FEA) to investigate the biomechanical characteristics of a cervical spine model after using the novel ATPRS intervertebral fusion system, providing insights into its application and potential refinement.

**Methods:** A finite element (FE) model of the C3-C7 lower cervical spine was established and validated. After two-level (C4-C6) anterior cervical discectomy and fusion (ACDF) surgery, FE models were constructed for the anterior cervical locked-plate (ACLP) internal fixation, the ATPS internal fixation, and the novel ATPRS intervertebral fusion system. These models were subjected to 75N axial force and 1.0 Nm to induce various movements. The range of motion (ROM) of the surgical segments (C4-C6), maximum stress on the internal fixation systems, and maximum stress on the adjacent intervertebral discs were tested and recorded.

**Results:** All three internal fixation methods effectively reduced the ROM of the surgical segments. The ATPRS model demonstrated the smallest ROM during flexion, extension, and rotation, but a slightly larger ROM during lateral bending. Additionally, the maximum bone-screw interface stresses for the ATPRS model during flexion, extension, lateral bending, and axial rotation were 32.69, 64.24, 44.07, 35.89 MPa, which were lower than those of the ACLP and ATPS models. Similarly, the maximum stresses on the adjacent intervertebral discs in the ATPRS model during flexion, extension, lateral bending, and axial rotation consistently remained lower than those in the ACLP and ATPS models. However, the maximum stresses on the cage and the upper endplate of the ATPRS model were generally higher.

**Conclusion:** Although the novel ATPRS intervertebral fusion system generally had greater endplate stress than ACLP and ATPS, it can better stabilize cervical three-column injuries and might reduce the occurrence of adjacent segment degeneration (ASD). Furthermore, further studies and improvements are necessary for the ATPRS intervertebral fusion system.

## Introduction

Based on prior research findings, it has been suggested that patients with severe cervical three-column injuries or those requiring multi-segment cervical decompression and reconstruction may not achieve sufficient stability by a solitary anterior approach fixation (Jack et al., 2017; Bayerl et al., 2019; Sethy et al., 2022). As a result, this inadequacy could potentially lead to increased postoperative complications and surgical failure (Bayerl et al., 2019). Some researchers argued that it is essential for these patients to undergo additional posterior surgery (Sethy et al., 2022). Nevertheless, the simultaneous utilization of anterior and posterior surgical approaches is linked to prolonged operative durations, increased trauma, and heightened risks (Wewel et al., 2019).

The ATPS method, as introduced by Koller et al., has demonstrated the capability to furnish enough stability through a sole anterior approach (Koller et al., 2008a). Previous investigations have revealed that the pull-out strength of ATPS surpasses that of cervical vertebral body screws (VBS) by 2.5 times, and it can yield comparable outcomes to the combined anterior and posterior surgeries (Koller et al., 2008b; Koller et al., 2009; Koller et al., 2010; Koller et al., 2015). Nevertheless, our research team, through a comprehensive series of studies on ATPS, has identified some ATPS limitations, including a heightened risk of screw insertion and technical complexity (Zhao et al., 2014; Zhao et al., 2016; Zhao et al., 2018; Li et al., 2022). Those all curtailed its widespread clinical applicability. So far, there only have been several reports on the clinical application of ATPS (Zhang et al., 2019; Pei et al., 2022). Consequently, we have undertaken enhancements to the ATPS technology, culminating in the proposal of the ATPRS technology (Zhang et al., 2022). In the sagittal plane, the head end of ATPRS is located on the axis of the pedicle; in the horizontal plane, the head end of ATPRS is positioned at the junction of the pedicle axis and the posterior edge of the vertebral body. Anatomically, the pedicle gradually widens from its narrowest point towards both sides, resembling an hourglass shape (Onibokun et al., 2009; Liu et al., 2010). The ATPRS can avoid passing the narrowest point of the cervical pedicle. So, the ATPRS technology, in theory, mitigates the risks associated with screw insertion. In our previous study, we have indicated the feasibility of the ATPRS intervertebral fusion system in the cervical spine (Ye et al., 2023). The system comprises three primary components: the insert positioned between the upper and lower vertebral bodies, the first screw connecting the upper vertebral body and the insert, and the second screw connecting the lower vertebral body and the insert. Besides, based on our previous radiographic study of the ATPRS fixed system, the screw hole positions in the cage were determined (Ye et al., 2023). However, there is a lack of direct biomechanical study. Therefore, the primary objective of this study is to conduct FEA to scrutinize and compare the alterations in range of motion (ROM) and stress distribution within the ACLP, the self-designed ATPS fixation system (ZL:201120445914), and the self-designed ATPRS intervertebral fusion system (ZL:2019202500823) in the context of a two-level discectomy decompression and bone graft fusion surgery (Zhao et al., 2018; Ye et al., 2023). This analysis can serve as a foundational basis for the subsequent design and application of the ATPRS intervertebral fusion system.

## Materials and methods

### Subject

At our hospital, we selected a healthy adult male volunteer, aged 28, with a height of 175 cm and a weight of 65 kg. This individual had no prior history of cervical spine trauma, diseases, surgeries, or related conditions. To ensure the participant’s suitability, frontal and lateral cervical spine radiographs were obtained, along with hyperextension and hyperflexion films, aimed at excluding cervical scoliosis, deformities, bone degradation, osteophytes, and cervical instability.

The study received the permission from the Ethics Committee of Ningbo No. 6 Hospital, affiliated with Ningbo University. Additionally, this study was based on image data and would not cause harm to the volunteers. It also would not disclose volunteer information. Therefore, by national legislation and institutional requirements, there was no need for participants or their legal guardians/next of kin to sign an informed consent form.

### Establishment of the intact FE model

Computed tomography (CT) images (64-channel scanner, Philips, Netherlands) of the volunteers’ entire C3-C7 spinal segments were recorded onto a CD in DICOM format. Subsequently, the CT image data of the lower cervical spine were imported into Mimics 21.0 software (Materialise, Belgium) for the construction of a 3D model. The bony structures of C3-C7 were isolated within a specific threshold range, and any necessary adjustments were made using the mask editing function. Ultimately, a preliminary 3D model encompassing the entire lower cervical spine from C3 to C7 was generated through the application of the 3D calculation function. This preliminary 3D model of the lower cervical spine was then imported into the reverse engineering software Geomagic Studio 2014 (3D Systems, Inc., United States) for surface refinement and optimization, and it was saved in STP format.

**FIGURE 1:**
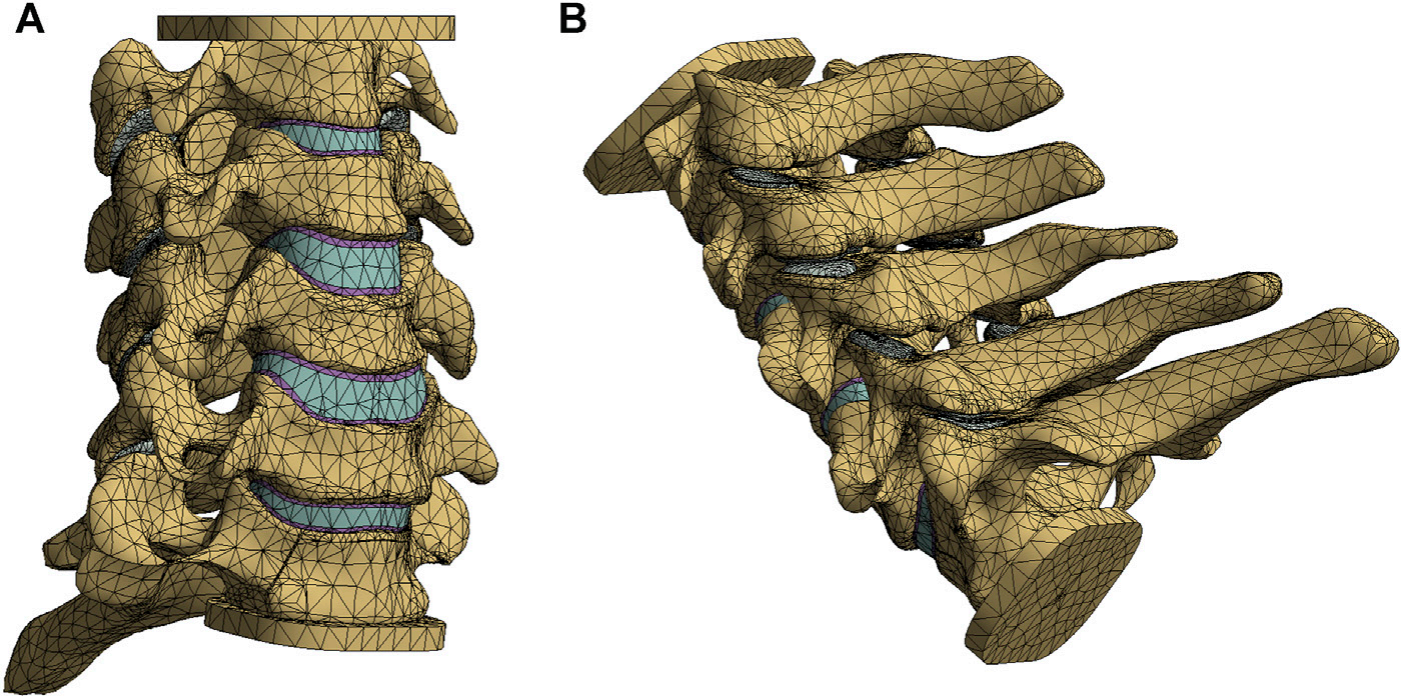
The intact finite element model of lower cervical spine: anterior oblique view **(A)**, posterior oblique view **(B)**.

The model, in STP format, was subsequently imported into Ansys Workbench 2019 (ANSYS, United States) for FE pre-processing. A meshing technique employing ten nodal cells was utilized. In Figure 1, the vertebral body comprised bone cancellous, bone cortical, and endplates, with the cortical bone set at a thickness of 0.5 mm, and the upper and lower endplates established at a thickness of 0.5 mm (Panjabi et al., 2001a). The intervertebral disc was divided into two distinct parts, the nucleus pulposus, and the annulus fibrosus, with the nucleus pulposus accounting for approximately 40% (Cai et al., 2020). Additionally, the ligaments incorporated in the model encompassed interspinous ligament, supraspinous ligament, anterior longitudinal ligament, posterior longitudinal ligament, transverse ligament, ligamentum flavum, and capsular ligaments. To optimize computational efficiency and convergence, a segmentation technique utilizing multiple two-node spring elements with only axial translational degrees of freedom was employed for the representation of the ligaments (Herron et al., 2020). While mesh-based ligament modeling may provide a more realistic appearance, it significantly hinders calculation convergence and demands extensive computational time. The spring element effectively achieves the same outcome, reducing computational time and enhancing model convergence. Material properties and element types for each tissue within the lower cervical spine model were referenced from previous literature (Table 1) (Zhao et al., 2018; Zhang et al., 2022; Manickam and Roy, 2022).

**TABLE 1:** Main material properties of the lower cervical finite element model.

Component	Young’s modulus (MPa)	Poisson’s ratio	Element type
Cortical bone	12000	0.29	Solid
Cancellous bone	450	0.29	Solid
Endplate	10	0.3	Solid
Cartilage	10	0.3	Solid
Nucleus pulposus	450	0.3	Solid
Annulus fibrosus	1	0.49	Solid
Screw	110,000	0.3	Solid
Plate	110,000	0.3	Solid
Cage	110,000	0.3	Solid

### Establishment of the two-level ACLP, ATPS, and ATPRS FE model

The complete lower cervical spine FE model was segmented in the ANSYS FEA software to create a three-column cervical injury model. Specifically, the ligaments (supraspinous, interspinous, bilateral facet joint capsules, ligamentum flavum, posterior longitudinal ligament) and the posterior part of the intervertebral disc between C4-C5 and C5-C6 were removed, leaving only a portion of the anterior longitudinal ligament.

**FIGURE 2:**
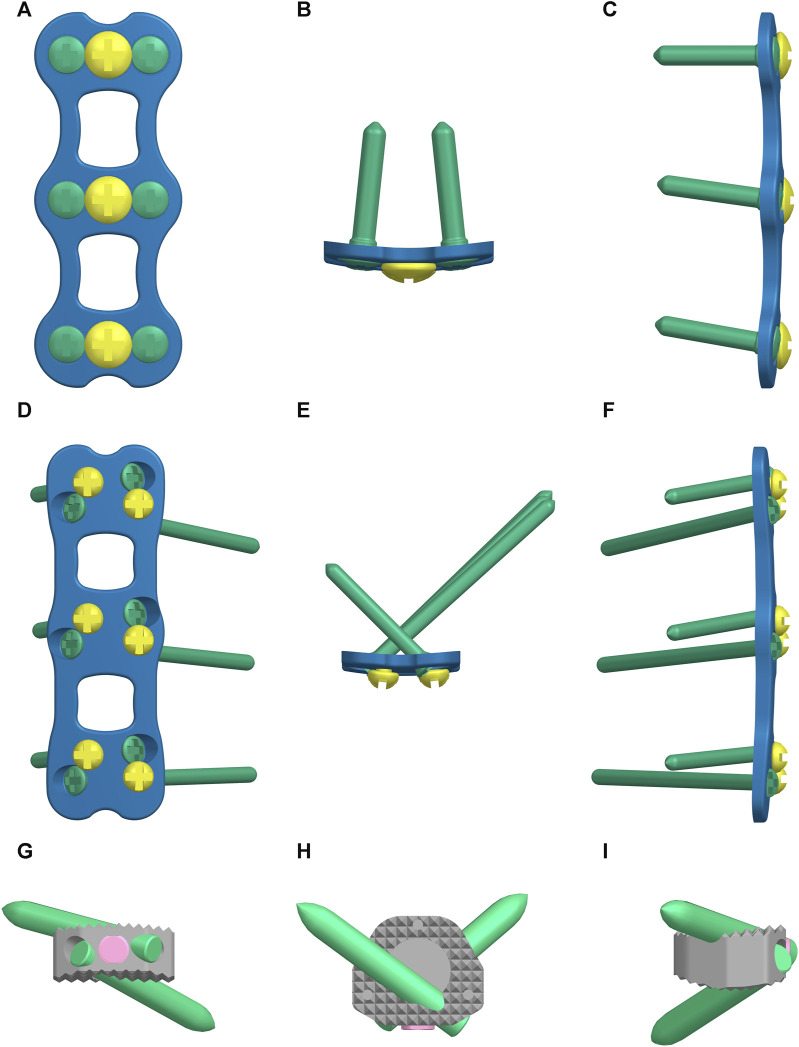
The three internal fixation methods: the anterior cervical locked-plate (ACLP) internal fixation **(A–C)**, the cervical anterior transpedicular screw (ATPS) internal fixation **(D–F)**, the cervical anterior transpedicular root screw (ATPRS) intervertebral fusion system **(G–I)**.

**FIGURE 3:**
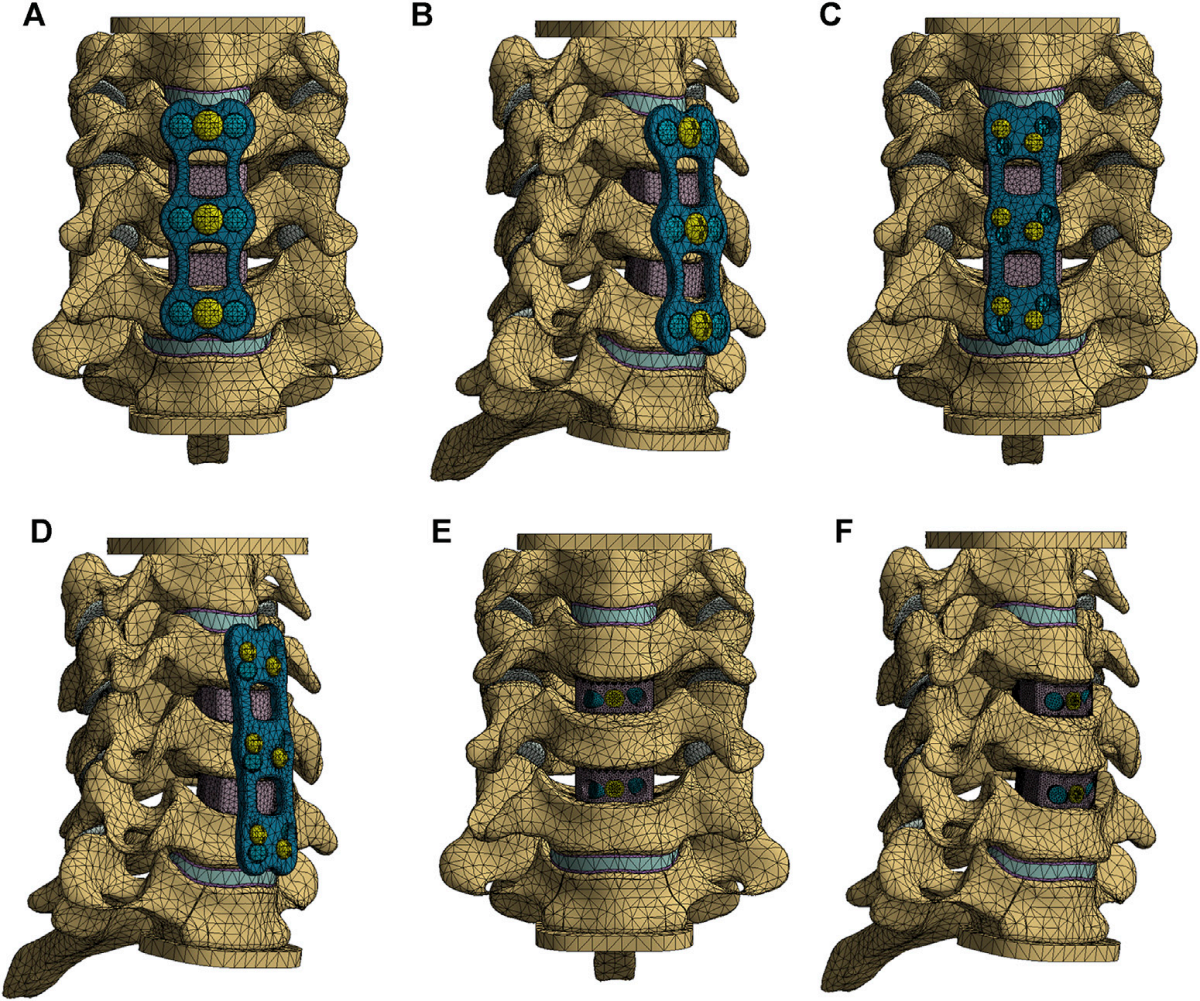
The finite element models of three internal fixation methods: the anterior cervical locked-plate (ACLP) internal fixation finite element model **(A, B)**; the cervical anterior transpedicular screw (ATPS) internal fixation finite element model **(C, D)**; the cervical anterior transpedicular root screw (ATPRS) intervertebral fusion system finite element model **(E, F)**.

Using Siemens NX1911 software (Siemens, Germany), we separately designed the ACLP internal fixation system, the ATPS internal fixation system, and the ATPRS intervertebral fusion system (Figure 2). Following the requirements of a two-level ACDF surgery, the C4-C5 and C5-C6 intervertebral discs were excised. The prepared three-column injury model was then combined with each internal fixation system to ensure a precise fit. For the ACLP internal fixation system, six vertebral screws with a length of 16 mm and a diameter of 4.0 mm were placed bilaterally. In the ATPS internal fixation system, three ATPS screws with a length of 30 mm and a diameter of 3.5 mm were inserted on one side, while on the other side, three vertebral screws with a length of 16 mm and a diameter of 3.5 mm were placed. In the ATPRS intervertebral fusion system, four ATPRS screws with a length of 22 mm and a diameter of 3.5 mm were inserted bilaterally. To simulate complete bony fusion, a rigid tied contact was defined between the screws, plates, cages, and vertebral bodies, which means that there is no relative displacement between screws, plates, cages, and vertebral bodies (Figure 3) (Lin et al., 2021).

### Loading and boundary conditions

Constraints were applied to the four FE models to keep the lower endplate of C7 completely fixed and C3 unconstrained. Based on previous literature, an axial pressure of 75 N was applied to the upper surface of the C3 vertebra to mimic the weight of the head (Zhang et al., 2022). A 1.0 Nm moment was loaded at the coupling point on the upper surface of the C3 vertebra to cause anterior flexion, posterior extension, lateral flexion, and axial rotational activity in the FE models (Lee et al., 2016; Liang et al., 2022). The ROM of cervical spine surgical segments, the stress distribution of screws, plates, fusion devices, upper endplate, and intervertebral discs were recorded for each model and analyzed comparatively.

## Results

### Model validation

**FIGURE 4:**
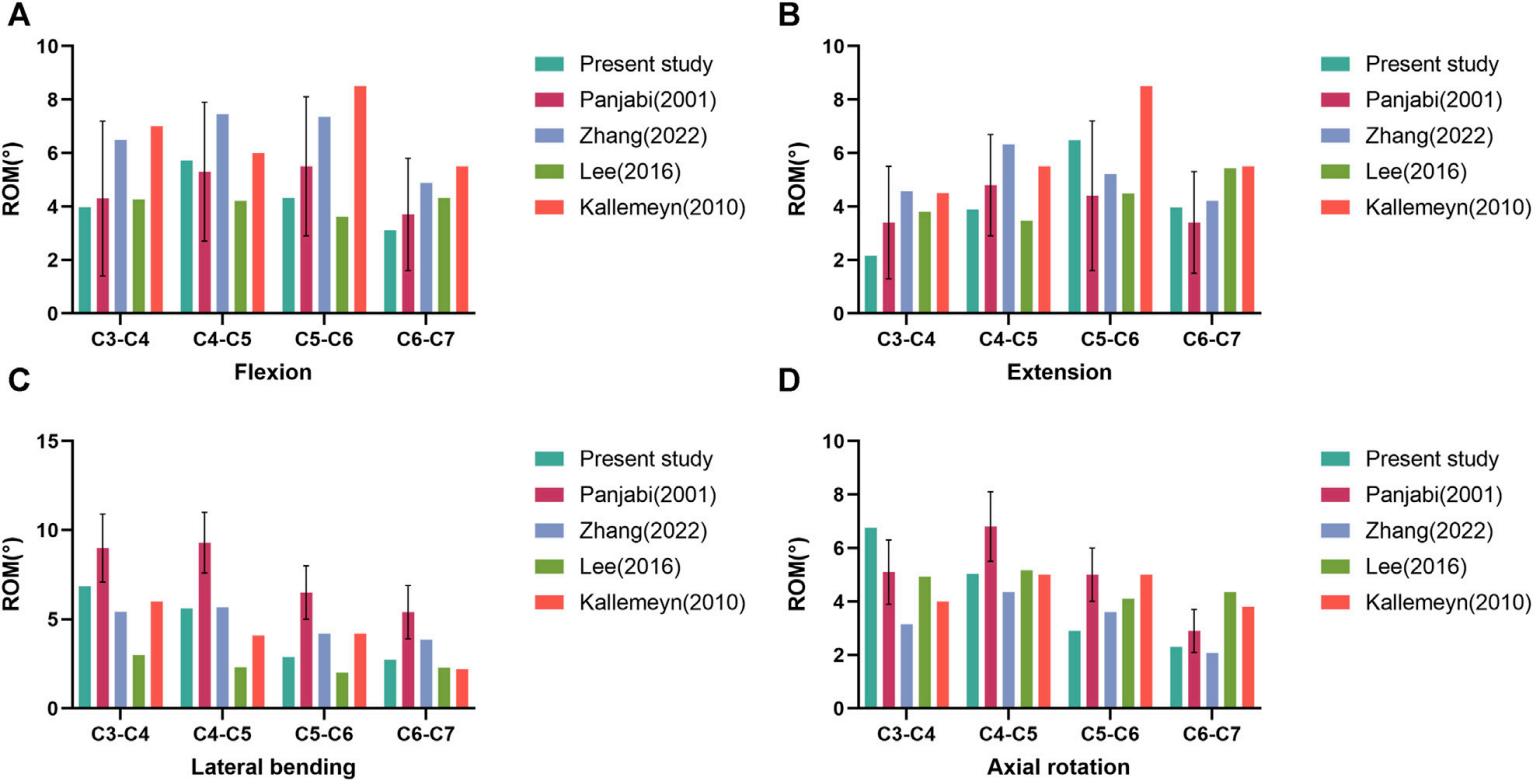
Comparison of the range of motion (ROM) of the intact three-dimensional finite element model of C3-C7 with the previous biomechanical studies. ROM in flexion **(A)**. ROM in extension **(B)**. ROM in lateral bending **(C)**. ROM in axial rotation **(D)**.

In this study, the intact lower cervical spine FE model has a total of 94245 elements and 164833 nodes, with realistic shape and good performance. In the intact lower cervical spine FE model, the ROMs at the levels of C3-C4, C4-C5, C5-6, and C6-7 were as follows: during flexion, they were 3.97°, 5.72°, 4.32°, and 3.11°, respectively; during extension, they were 2.16°, 3.89°, 6.48°, and 3.96°, respectively; during lateral bending, they were 6.86°, 5.62°, 2.90°, and 2.74°, respectively; and during axial rotation, they were 6.76°, 5.04°, 2.90°, and 2.30°, respectively. In Figure 4, the ROMs of the model were in high agreement with the previous study, which indicates the validity of the present study model (Panjabi et al., 2001b; Kallemeyn et al., 2010; Lee et al., 2016; Zhang et al., 2022). And this FE model could be used in the later study.

### ROMs of the cervical spine surgical segments

**FIGURE 5:**
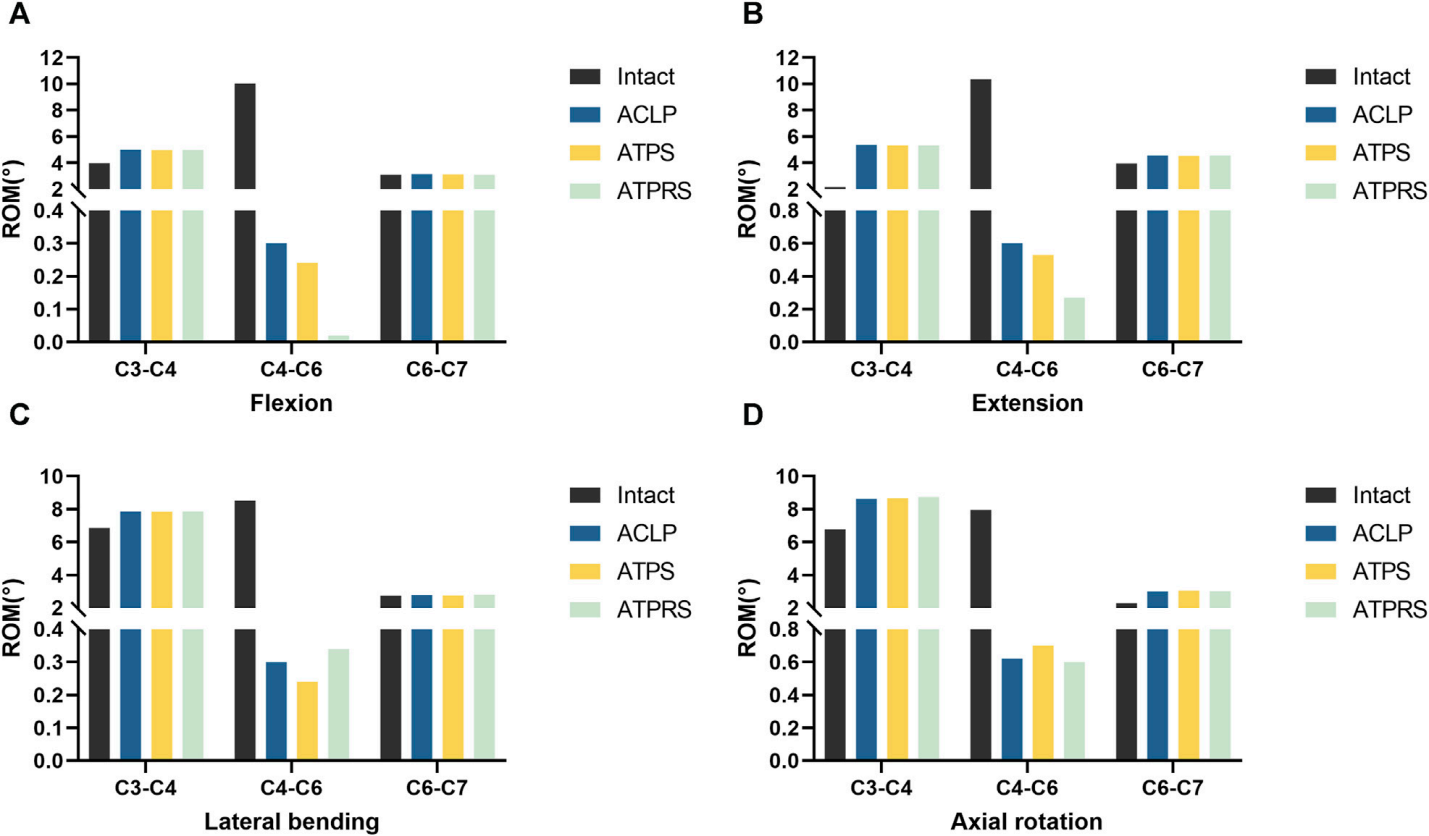
Comparisons of the range of motion (ROM) of the surgical segments (C4–C6) between the intact model and three internal fixation models (ACLP, ATPS, ATPRS): ROM in flexion **(A)**. ROM in extension **(B)**. ROM in lateral bending **(C)**. ROM in axial rotation **(D)**.

As illustrated in Figure 5, the ROMs for flexion, extension, lateral bending, and axial rotation in the intact surgical segments (C4-C6) were measured at 10.04°, 10.37°, 8.52°, and 7.94°, respectively. Following the two-level corpectomy procedure, the three internal fixation methods substantially reduced the ROMs in C4-C6. Specifically, the ATPRS model demonstrated a 93.3% reduction in mobility in flexion, while the ATPS model showed a 20.0% reduction compared to the ACLP model. In extension, the ATPRS model exhibited a 53.3% reduction, and the ATPS model showed an 11.6% reduction compared to the ACLP model. In lateral bending, the ATPRS intervertebral fusion system increased mobility by 13.3% compared to the ACLP model but the ATPS model decreased mobility by 20.0% compared to the ACLP model. Regarding axial rotation, the ATPRS intervertebral fusion system decreased mobility by 3.2% compared to the ACLP model, while the ATPS model increased it by 12.9%. Furthermore, all three internal fixation methods resulted in increased ROMs in the C3-C4 segment due to the fixed fusion of the surgical segments.

### Stress of internal fixation system

As depicted in Table 2, when subjected to a load of 1.0 Nm, the ATPRS model exhibited maximum bone-screw interface stresses of 32.69 MPa, 64.24 MPa, 44.07 MPa, and 35.89 MPa during flexion, extension, lateral bending, and axial rotation, respectively. These values were all lower than those of the ATPS model and ACLP model. The ATPS model exhibited higher maximum bone-screw interface stresses than the ACLP model in lateral bending and axial rotation but lower stresses in flexion and extension. In terms of plate stresses, the maximum von Mises stresses were predominantly at the screw-plate interface. In flexion, extension, and lateral bending, the ATPS model has higher plate stresses than the ACLP model, except for axial rotation (Table 3).

**TABLE 2:** The maximum von Mises stresses at the bone-screw interface of the three internal fixation models (ACLP, ATPS, ATPRS) (MPa).

	ACLP	ATPS	ATPRS
Flexion	53.72	47.77	32.69
Extension	73.89	72.26	64.24
Lateral bending	52.03	129.62	44.07
Axial rotation	46.66	60.94	35.89

**TABLE 3:** The maximum von Mises stresses on the plate of the three internal fixation models (ACLP, ATPS, ATPRS) (MPa).

	ACLP	ATPS	ATPRS
Flexion	53.92	58.00	-
Extension	79.86	107.99	-
Lateral bending	80.34	93.26	-
Axial rotation	61.41	54.99	-

**FIGURE 6:**
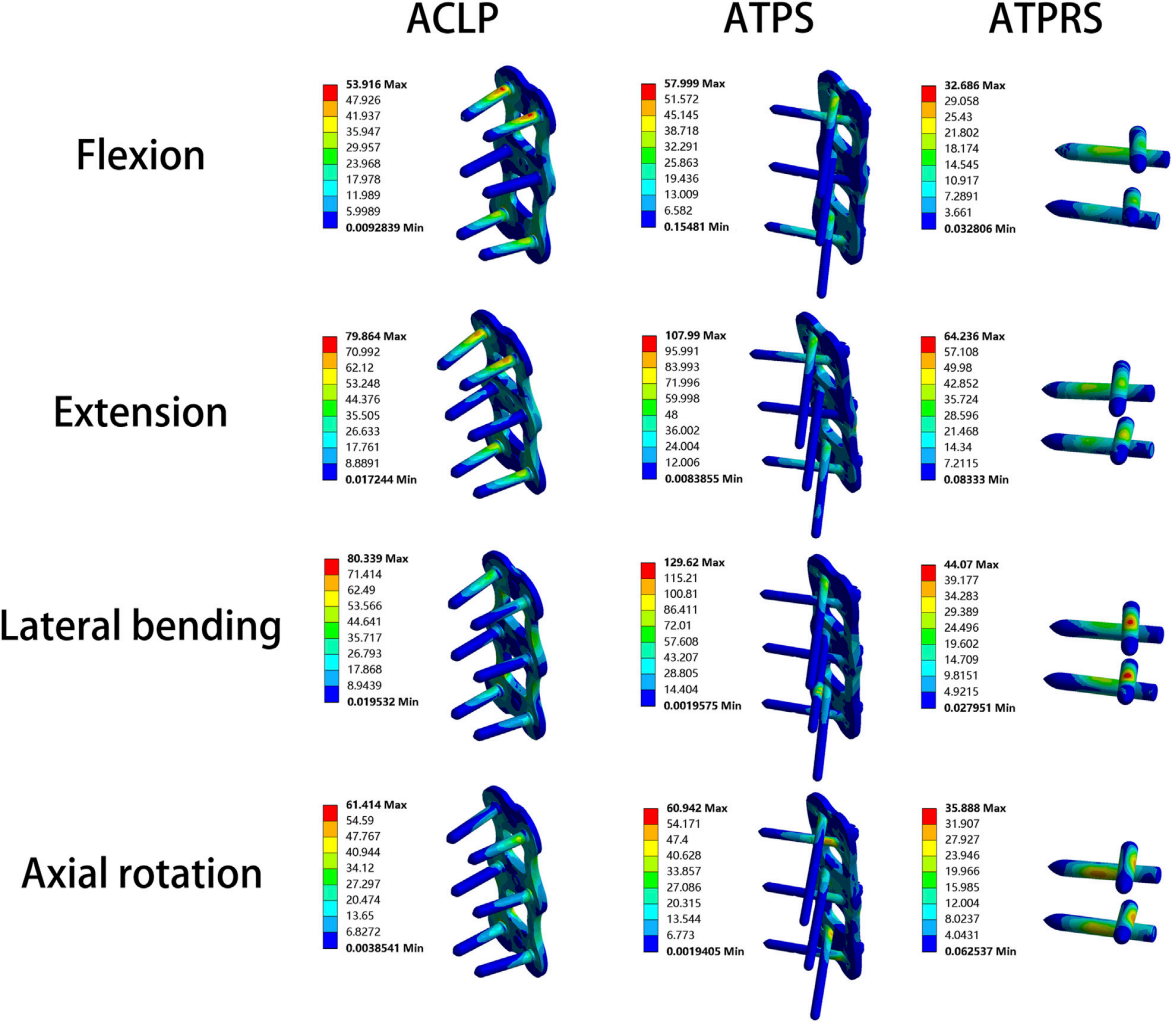
Stress cloud map of the screw–bone interface and plate of three internal fixation models (ACLP, ATPS, ATPRS) in flexion, extension, lateral bending, and axial rotation.

**FIGURE 7:**
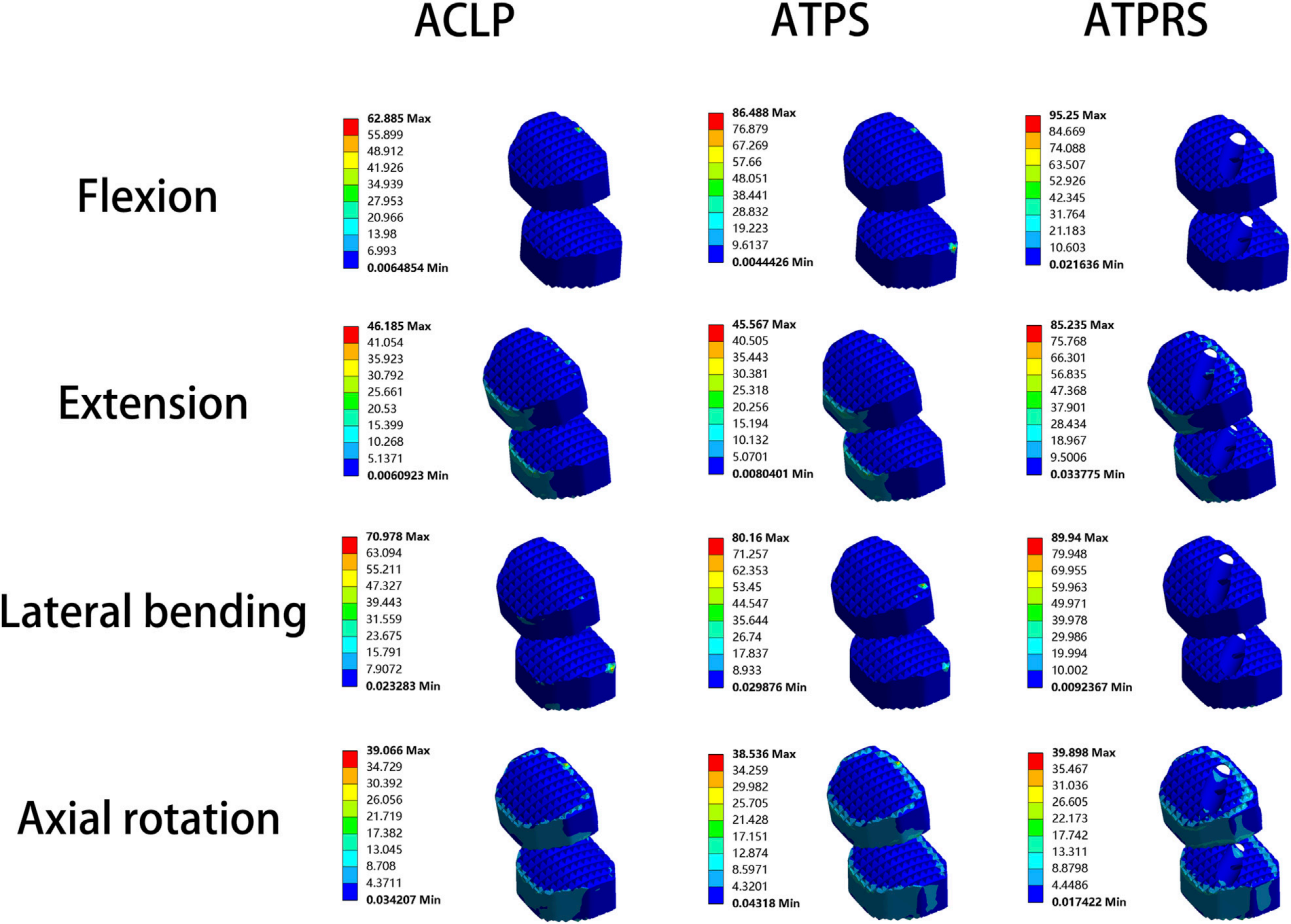
Stress cloud map of the cage of three internal fixation models (ACLP, ATPS, ATPRS) in flexion, extension, lateral bending, and axial rotation.

Regarding the cage stresses (Table 4), at the C4-C5 level in the ACLP model, the maximum von Mises stress values for flexion, extension, lateral bending, and axial rotation were 62.89, 29.72, 37.88, and 38.28 MPa, respectively. For the ATPS model, these respective values were 62.37, 30.98, 80.16, and 38.26 MPa, while for the ATPRS model, they were 90.02, 85.24, 50.19, and 39.90 MPa. At the C5-C6 level, in the ACLP model, the maximum von Mises stress values for flexion, extension, lateral bending, and axial rotation were 50.57, 46.19, 70.98, and 39.07 MPa, respectively. For the ATPS model, these respective values were 86.49, 45.57, 70.79, and 38.54 MPa, while for the ATPRS model, they were 95.25, 59.26, 89.94, and 23.85 MPa. Stress distribution maps for the internal fixation system (screws, plates, cage) under flexion, extension, lateral bending, and axial rotation conditions are illustrated in Figures 6, 7.

**TABLE 4:** The maximum von Mises stresses on the cages of the three internal fixation models (ACLP, ATPS, ATPRS) (MPa).

		ACLP	ATPS	ATPRS
C4-C5	Flexion	62.89	62.37	90.02
	Extension	29.72	30.98	85.24
	Lateral bending	37.88	80.16	50.19
	Axial rotation	38.28	38.26	39.90
C5-C6	Flexion	50.57	86.49	95.25
	Extension	46.19	45.57	59.26
	Lateral bending	70.98	70.79	89.94
	Axial rotation	39.07	38.54	23.85

### Stress on the upper endplate

In Table 5, for the upper endplate of C5, the intact model exhibited maximum stresses were 8.91, 11.08, 12.24, and 6.03 MPa during flexion, extension, lateral bending, and axial rotation, respectively; In the ACLP model, these values were 17.18, 41.50, 53.28, and 26.82 MPa, respectively; In the ATPS model, these values were 50.12, 44.35, 78.40, and 30.29 MPa, respectively; In the ATPS model, these values were 116.55, 98.52, 54.85, and 36.38 MPa, respectively. For the upper endplate of C6, the intact model exhibited maximum stresses were 8.61, 10.55, 8.99, and 5.16 MPa during flexion, extension, lateral bending, and axial rotation, respectively; In the ACLP model, these values were 18.15, 53.30, 37.34, and 47.04 MPa, respectively; In the ATPS model, these values were 18.05, 55.36, 40.23, and 48.53 MPa, respectively; In the ATPS model, these values were 19.34, 60.29, 52.69, and 24.91 MPa, respectively.

**TABLE 5:** The maximum von Mises stresses on the upper endplate of the four models (INTACT, ACLP, ATPS, ATPRS) (MPa).

		Intact	ACLP	ATPS	ATPRS
C5	Flexion	8.91	17.18	50.12	116.55
	Extension	11.08	41.50	44.35	98.52
	Lateral bending	12.24	53.28	78.40	54.85
	Axial rotation	6.03	26.82	30.29	36.38
C6	Flexion	8.61	18.15	18.05	19.34
	Extension	10.55	53.30	55.36	60.29
	Lateral bending	8.99	37.34	40.23	52.69
	Axial rotation	5.16	47.04	48.53	24.91

### Stress on the intervertebral disc

**FIGURE 8:**
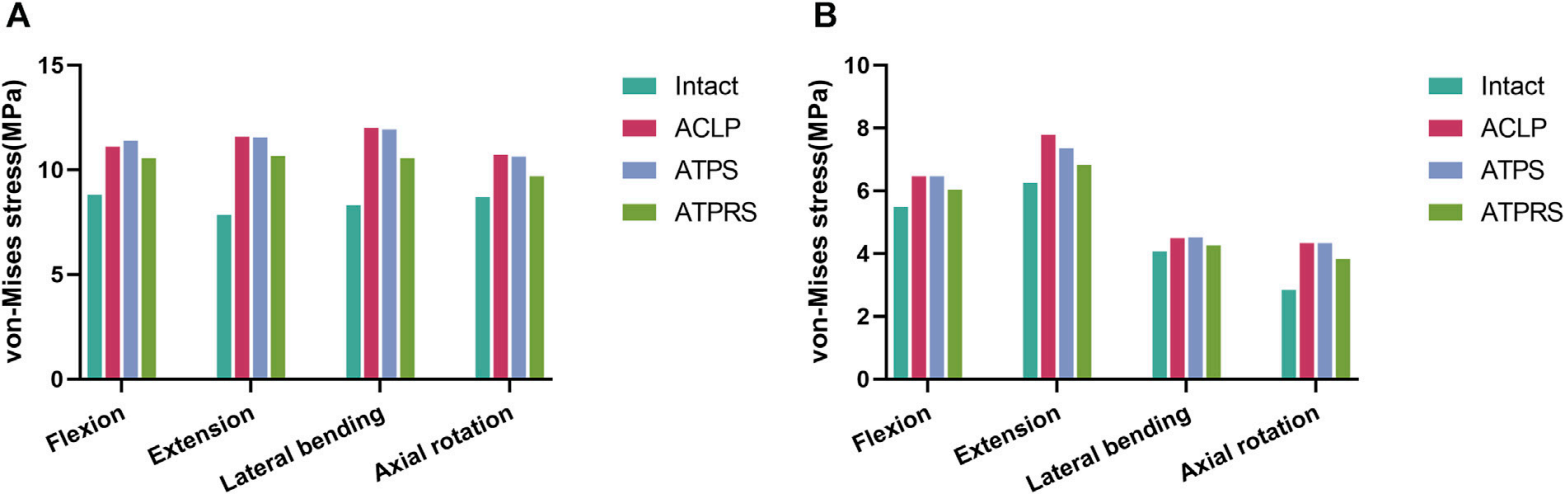
Comparisons of the von Mises stress of the adjacent intervertebral disc between the intact model and three internal fixation models (ACLP, ATPS, ATPRS) in flexion, extension, lateral bending, and axial rotation: the stress on the C3-C4 intervertebral disc **(A)**, the stress on the C6-C7 intervertebral disc **(B)**.

**FIGURE 9:**
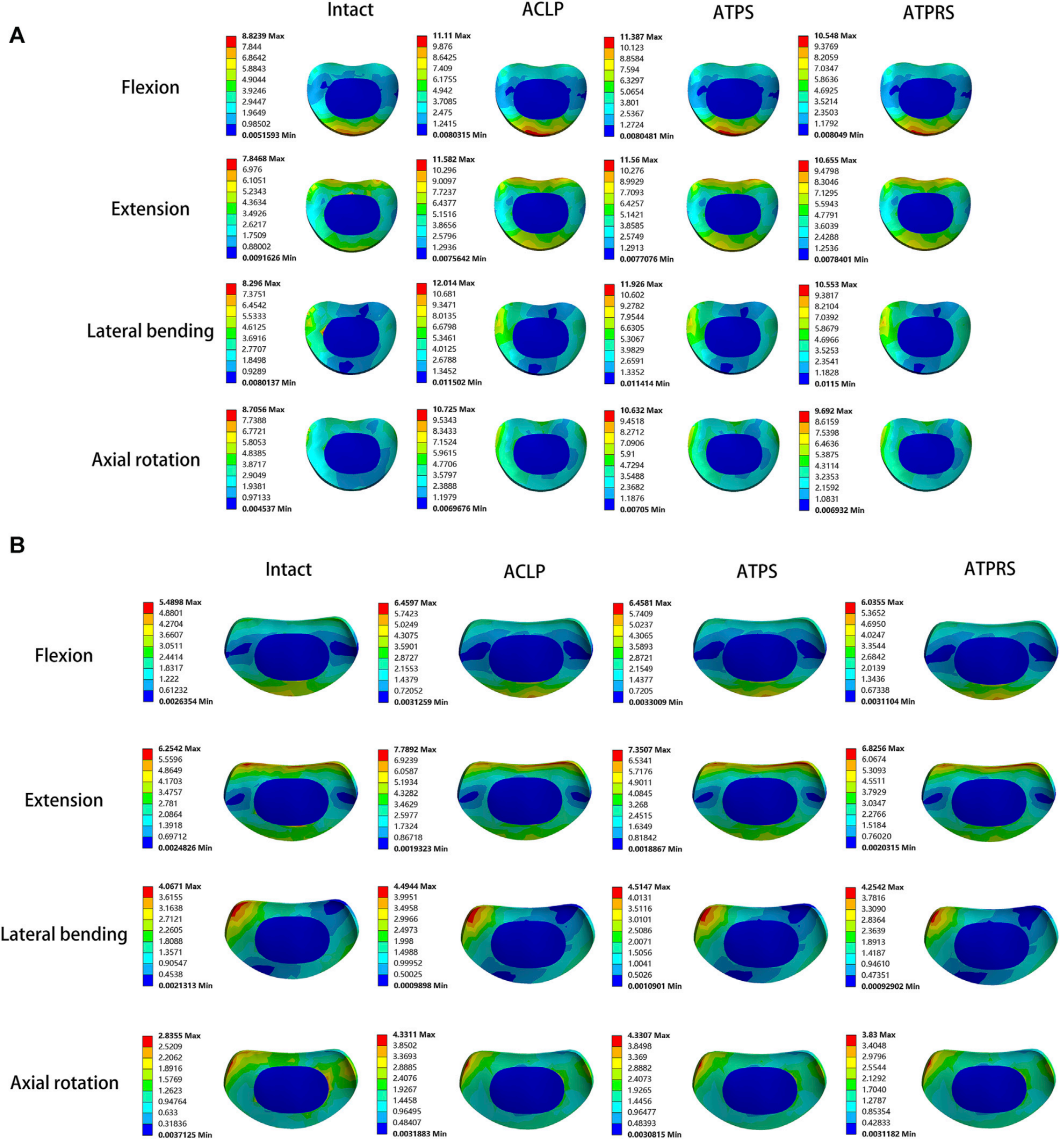
Stress cloud map of the adjacent intervertebral disc of the intact model and three internal fixation models (ACLP, ATPS, ATPRS) in flexion, extension, lateral bending, and axial rotation: the stress cloud map of the C3-C4 intervertebral disc **(A)**, the stress cloud map of the C6-C7 intervertebral disc **(B)**.

Figure 8 illustrates the maximum von Mises stresses of the C3-C4 and C6-C7 intervertebral discs. For the C3-C4 intervertebral disc, the maximum von Mises stresses in the intact model during flexion, extension, lateral bending, and axial rotation were 8.82, 7.85, 8.30, and 8.71 MPa, respectively. In the ACLP model, these values were 11.11, 11.58, 12.01, and 10.73 MPa, while in the ATPS model, they were 11.39, 11.56, 11.93, and 10.63 MPa, and in the ATPRS model, they were 10.55, 10.66, 10.55, and 9.69 MPa, respectively. As for the C6-C7 intervertebral disc, the maximum von Mises stresses in the intact model during flexion, extension, lateral bending, and axial rotation were 5.49, 6.25, 4.07, and 2.84 MPa, respectively. In the ACLP model, these values were 6.46, 7.79, 4.49, and 4.33 MPa, while in the ATPS model, they were 6.46, 7.35, 4.51, and 4.33 MPa, and in the ATPRS model, they were 6.04, 6.83, 4.25, and 3.83 MPa, respectively. The stress distribution of the C3-C4 and C6-C7 intervertebral discs is depicted in Figure 9.

## Discussion

FEA is a valuable method for investigating biomechanics in models. The primary steps involve establishing and validating the FE model. Once validated, the model can effectively accommodate various constraints, loading conditions, and material properties for different tests conducted by researchers (Wang et al., 2023). Consequently, this analysis method has been widely utilized in cervical spine biomechanics research (Lin et al., 2021; Zhang et al., 2022; Liang et al., 2022). In this study, we used cervical spine CT imaging data from a healthy adult male to rebuild the intact FE model of the lower cervical spine, and FE models of ACLP, ATPS, and ATPRS internal fixation methods were simultaneously established. This study aims to explore the biomechanical characteristics of the cervical spine FE model after using the self-designed ATPRS intervertebral fusion system. We applied 75N axial pressure and 1.0 Nm to the intact model, and the ROMs in flexion, extension, lateral bending, and axial rotation aligned with findings from previous studies (Figure 4), demonstrating the reliability of the FE model used in this study.

Through the analysis of the tests, all three internal fixation methods effectively reduced the mobility of the surgical segment. Moreover, the FE model of the ATPRS intervertebral fusion system exhibited the most substantial reduction in mobility for the C4-C6 segment during flexion, extension, and rotation. However, compared with the ACLP model and ATPS model, the ROM of the C4-C6 segment showed slightly greater during lateral bending in the ATPRS model. These collective findings suggested that the ATPRS intervertebral fusion system can provide adequate stability for cervical three-column injuries and was superior to the ATPS and ACLP internal fixation systems. Adjacent segment degeneration (ASD) is a common complication after cervical spine surgery, with its risk factors including a compensatory increase in the ROM of non-fused segments (Wong et al., 2020). Previous studies have indicated that the loss of mobility in the fused segment leads to a compensatory increase in the mobility of the non-fused segments after surgery (Hua et al., 2020; Choi et al., 2021), consistent with our present study. Specifically, we observed a more obvious compensatory increase in ROM for C3-C4, while the increase in ROM for C6-C7 was less significant. This result may indicate a higher risk of degeneration in the upper adjacent segments.

We observed that the maximum stress at the bone-screw interface in the ATPRS model was consistently lower than that in the ACLP and ATPS models, regardless of the type of movement (Table 2). This finding may suggest a reduced risk of postoperative complications such as screw loosening or fracture in the ATPRS intervertebral fusion system (Lin et al., 2021). Additionally, during lateral bending and axial rotation, the maximum stress at the bone-screw interface in the ATPS model was 129.62 and 60.94 MPa, respectively, which was higher than that of the ATPRS model and the ACLP model. This difference may be attributed to the asymmetric design of the ATPS internal fixation system, resulting in greater stress on the side with deeper insertion. However, the maximum stress at the bone-screw interface in the ATPS model was lower than that in the ACLP model during flexion and extension. The stress cloud map indicated that the maximum stress on the screws and plates mainly occurs at the contact area between the screws and plates, consistent with previous studies (Li et al., 2022) (Figure 6). In Table 3, the maximum stress on the plate in the ATPS model was greater than that in the ACLP model during flexion, extension, and lateral bending, but lower during axial rotation.

Cage subsidence, a common complication after ACDF surgery, occurs at a rate of approximately 20.0% (Noordhoek et al., 2018). While mild cage subsidence generally does not lead to corresponding symptoms, severe cage subsidence may result in foraminal stenosis, local kyphotic deformity, recurrent pain symptoms, and even reoperation (Brenke et al., 2015; Pinter et al., 2022; Pinter et al., 2023). The stress on the vertebral endplate to some extent reflects the likelihood of cage subsidence and greater endplate stress often leads to a higher risk of cage subsidence (Shen et al., 2022). In our study, the upper endplate stresses of the three internal fixation models all exceed that of the intact model. Specifically, in most cases, the upper endplate stresses of the ATPRS model were greater than that of the ACLP model and the ATPS model. This might imply a potentially greater risk of cage subsidence in the ATPRS intervertebral fusion system (Ahn et al., 2023). Previous studies have similarly indicated that the rate of subsidence with a plate and cage combined is lower than that with a standalone cage and screws (Xu et al., 2020; Dhar et al., 2023). In terms of cage stress, during flexion, stress was mainly concentrated on the anterior side of the cage; during extension, stress was primarily concentrated on the posterior side of the cage; during lateral bending, stress was mainly concentrated on both sides of the cage; during compression, stress was mainly distributed around the edges of the cage. Additionally, the screw holes in the ATPRS model’s cage were also areas of stress concentration. During the processes of flexion, extension, and lateral bending, the maximum stress on the cage in the ATPRS model was greater than that in the ACLP and ATPS models, except for the maximum stress on the cage at C4-C5 during lateral bending, where the ATPRS model was lower than the ATPS model. The possible reason for these results was that the plate shares some of the force, leading to less pressure on the fusion cage in the ACLP and ATPS models during movement. However, during axial rotation, the maximum stress on the fusion cage in the ATPRS model at C5-C6 was lower than that in the ACLP and ATPS models. This difference may be due to the direction of the torque during rotational movement, which was perpendicular to the long axis of the cervical spine and did not directly act on the fusion cage.

In the stress distribution map (Figure 9), the stresses on the adjacent intervertebral discs in the four models were observed to be concentrated at the anterior edge, posterior edge, posterior lateral edge, and posterior lateral edge during flexion, extension, lateral bending, and axial rotation, respectively. This spatial distribution aligned with the primary compression positions of the intervertebral discs during cervical spine movement in various directions. Additionally, the stress on the intervertebral discs in the three postoperative internal fixation models consistently surpassed that in the intact model during each type of motion. Similar findings have been reported in related research (Eck et al., 2002; Wu et al., 2019; Zhang et al., 2022). For instance, Eck et al. (Eck et al., 2002)demonstrated a notable increase in pressure on the adjacent intervertebral discs following cervical spine fusion surgery based on cadaveric cervical spine studies. Similarly, Wu et al. (Wu et al., 2019)identified an increase in stress on the adjacent intervertebral discs after the insertion of a cage at C3-C4 and C5-C6 in a FE model of the cervical spine. Notably, the stress on the adjacent intervertebral discs is closely associated with the development of adjacent segment degeneration (ASD) (Arun et al., 2009). In Figure 8, it was evident that the maximum stresses on the adjacent intervertebral discs in the ATPRS model during flexion, extension, lateral bending, and axial rotation were 10.55, 10.66 MPa, 10.55, and 9.69 MPa in C3-C4 and were 6.04, 6.83, 4.25, and 3.83 MPa in C6-C7. Those values consistently remained lower than those in the ACLP and ATPS models. This observation may suggest a reduced risk of ASD occurrence when utilizing the ATPRS intervertebral fusion system, thereby retaining the advantages associated with a zero-profile fusion cage (Li et al., 2020; Ahn et al., 2023).

Several limitations are present in this study. Firstly, the FE model, due to its omission of the influence of neck muscles, cannot fully replicate physiological cervical spine movement. Consequently, the analytical results may exhibit discrepancies with actual clinical outcomes. And vitro biomechanical studies of the ATPRS intervertebral fusion system need to be improved in further study. Secondly, in this study, the screw model was simplified and lacked threads to ensure better convergence of the FE model. Thirdly, the FE model was reconstructed from cervical spine CT images of a healthy adult, without accounting for conditions such as osteoporosis, osteophytes, and intervertebral disc degeneration. As such, its representativeness is limited, warranting further comprehensive investigation.

In conclusion, although the novel ATPRS intervertebral fusion system generally led to higher endplate stress and may increase the risk of cage subsidence compared to ACLP and ATPS, it can provide more significant benefits in terms of reconstructing the stability of cervical spine three-column injury and preventing ASD. Additionally, further research and improvement of the ATPRS intervertebral fusion system are still required, including *in vitro* animal or cadaveric cervical spine experiments.

## Data Availability

The original contributions presented in the study are included in the article/Supplementary material, further inquiries can be directed to the corresponding author.
